# Correction: Pre-Treatment Ferritin Level and Alveolar-Arterial Oxygen Gradient Can Predict Mortality Rate Due to Acute/Subacufte Interstitial Pneumonia in Dermatomyositis Treated by Cyclosporine A/Glucocorticosteroid Combination Therapy: A Case Control Study

**DOI:** 10.1371/journal.pone.0101489

**Published:** 2014-06-23

**Authors:** The PLOS ONE Staff

## Notice of Republication

This article was republished on June 5, 2014, to correct an error in the title of the article that was introduced during the typesetting process. The correct title of the article is “Pre-Treatment Ferritin Level and Alveolar-Arterial Oxygen Gradient Can Predict Mortality Rate Due to Acute/Subacute Interstitial Pneumonia in Dermatomyositis Treated by Cyclosporine A/Glucocorticosteroid Combination Therapy: A Case Control Study.” Please download this article again to view the correct version. The originally published, uncorrected article and the republished, corrected article are provided here for reference.

## Supporting Information

File S1 Originally published, uncorrected article.
(PDF)

File S2 Republished corrected article. Originally published, uncorrected article.
(PDF)
