# Supplementary material for: Correction: Pre-Treatment Ferritin Level and Alveolar-Arterial Oxygen Gradient Can Predict Mortality Rate Due to Acute/Subacufte Interstitial Pneumonia in Dermatomyositis Treated by Cyclosporine A/Glucocorticosteroid Combination Therapy: A Case Control Study
Source: PLoS One. 2014 Jun 23;9(6):e101489. doi: 10.1371/journal.pone.0101489 (PMC4067414; doi:10.1371/journal.pone.0101489)
Supplement: File S1 — Originally published, uncorrected article. (PDF) [file pone.0101489.s001.pdf]

PLOS | ONE

Pre-Treatment Ferritin Level and Alveolar-Arterial Oxygen Gradient Can Predict Mortality Rate Due to Acute/Subacufte Interstitial Pneumonia in Dermatomyositis Treated by Cyclosporine A/ Glucocorticosteroid Combination Therapy: A Case Control Study

Kentaro Isoda, Tohru Takeuchi*, Takuya Kotani, Kenichiro Hata, Takeshi Shoda, Takaaki Ishida, Shuzo Yoshida, Yuko Kimura, Shigeki Makino, Toshiaki Hanafusa

Department of Internal Medicine (I), Osaka Medical College, Takatsuki, Osaka, Japan

Abstract

Background: Acute/subacute interstitial pneumonia in dermatomyositis (DM-A/SIP) is a disease associated with a poor prognosis that resists treatment with glucocorticosteroids (GC) and progresses rapidly in a period of weeks to months to death. We retrospectively studied outcomes, prognostic factors, and their relations with survival rate in patients with DM-A/SIP treated with early cyclosporine A (CSA)/GC combination therapy and 2-hour postdose blood concentration monitoring.

Methods: This study comprised 32 DM-A/SIP patients who were simultaneously treated with CSA and prednisolone. Clinical and laboratory findings were compared between those who died due to DM-A/SIP and those surviving 24 weeks after beginning of therapy. Prognostic factors were extracted, and their relations with the survival rate were evaluated.

Results: Of the 32 DM-A/SIP patients, 25 survived, 5 died of DM-A/SIP, and 2 died of infections. In those who died due to DM-A/SIP, ferritin level and the alveolar-arterial oxygen gradient were significantly increased compared with the survivors ($P<0.001$ and $P=0.002$, respectively). Multivariate analyses showed that ferritin and alveolar-arterial oxygen gradient were independent prognostic factors of poor outcome. The survival rate 24 weeks after beginning of treatment was significantly lower in those with a ferritin level of ≥600 ng/ml and alveolar-arterial oxygen gradient of ≥45 Torr ($P<0.001$ and $P<0.001$, respectively). All patients with both prognostic factors died, and the outcome was significantly poorer in these patients than in those with one or neither of the prognostic factors ($P<0.001$).

Conclusions: We identified pre-treatment high serum ferritin level and high alveolar-arterial oxygen gradient as poor prognostic factors in DM-A/SIP patients undergoing early CSA/GC combination therapy and showed that the outcomes were poor in patients with both factors.

Citation: Isoda K, Takeuchi T, Kotani T, Hata K, Shoda T, et al. (2014) Pre-Treatment Ferritin Level and Alveolar-Arterial Oxygen Gradient Can Predict Mortality Rate Due to Acute/Subacufte Interstitial Pneumonia in Dermatomyositis Treated by Cyclosporine A/Glucocorticosteroid Combination Therapy: A Case Control Study. PLoS ONE 9(2): e89610. doi:10.1371/journal.pone.0089610

Editor: Masataka Kuwana, Keio University School of Medicine, Japan

Received October 29, 2013; **Accepted** January 22, 2014; **Published** February 21, 2014

Copyright: © 2014 Isoda et al. This is an open-access article distributed under the terms of the Creative Commons Attribution License, which permits unrestricted use, distribution, and reproduction in any medium, provided the original author and source are credited.

Funding: The authors have no support or funding to report.

Competing Interests: The authors have declared that no competing interests exist.

* E-mail: t-takeuchi@poh.osaka-med.ac.jp

Introduction

Dermatomyositis (DM) is an autoimmune inflammatory muscle disorder that symmetrically affects primarily the proximal limb, neck, and pharyngeal muscles and is accompanied by a characteristic rash such as Gottron's papules and heliotrope eyelids. It is often complicated by interstitial pneumonia (IP) and is classified into chronic IP and acute/subacute IP (A/SIP) on the basis of clinical course. DM-A/SIP is a disease associated with a poor prognosis that resists treatment with glucocorticosteroids (GC) and progresses rapidly in a period of weeks to months to death [1,2]. Recently, early intervention with a combination of GC and a calcineurin inhibitor such as cyclosporine A (CSA) and tacrolimus and this combination plus intravenous pulse cyclophosphamide therapy (IVCY) was reported to improve the prognosis of DM-A/SIP [3–6]. However, there are still many patients who cannot be saved even with these regimens. Early additional immunosuppressant therapy is necessary for such patients, but an accurate prognosis or evaluation of the severity is difficult before the beginning of treatment.

Clinical amyopathic DM (C-ADM), autoantibodies such as anti-aminoacyl tRNA synthetase (ARS) and anti-melanoma

differentiation-associated gene 5 (MDA5) antibodies (Ab), high-resolution computed tomography findings such as ground-glass attenuation and reticular opacity, and a decrease in the diffusing capacity on respiratory function tests have been reported as findings related to the severity and prognosis of DM-IP [6–12]. Recently, among serum biomarkers, KL-6 and ferritin have been reported to be useful prognostic factors for DM-A/SIP [9,13,14]. However, prognostic factors of DM-A/SIP are difficult to analyse because, in addition to these factors, the disease type of IP, time until the beginning of treatment, and therapeutic strategy are also known to affect the outcome.

We retrospectively investigated the relations of blood gas analysis results and serum markers such as KL-6 and ferritin before the beginning of treatment with outcome in DM-A/SIP patients who underwent early CSA/GC combination therapy and 2-hour postdose blood concentration (C2) monitoring. We also evaluated the relations of prospective prognostic factors with outcome in DM-A/SIP.

Materials and Methods

Study design

Participants in this retrospective study comprised 41 patients with DM-A/SIP admitted to Osaka Medical College Hospital during the period from March 2004 to April 2012. DM was diagnosed according to the criteria of Bohan and Peter [15,16]. Clinical amyopathic DM was diagnosed according to the criteria proposed by Sontheimer and Gerami et al. [17,18]. IP was evaluated with chest radiography and chest HRCT. A/SIP was defined as IP with a respiratory condition, laboratory findings, arterial gas findings, and HRCT images rapidly exacerbating in a period of days to 3 months after disease onset [3,19]. After excluding 1 patient with ANCA-related vasculitis, 1 with scleroderma, 6 who had received high-dose GC therapy, steroid pulse therapy, or immunosuppressant therapy before admission to our hospital, and 1 in whom CSA could not be used due to an adverse event (liver dysfunction), 32 patients were analysed. The patients were divided into those who were still alive and those who had died by 24 weeks after the beginning of CSA/GC combination therapy. Those who died were divided into those who died due to DM-A/SIP and those who died due to causes other than deterioration of IP, such as infection. Clinical and laboratory findings were compared between the survivors and those who had died due to DM-A/SIP, prognostic factors were extracted, and their relations with survival rate were evaluated.

Ethics Statement

This study was conducted in accordance with the Declaration of Helsinki and its amendments and was approved by the Osaka Medical College and Faculty of Medicine Ethics Committee. Written informed consent was obtained from each patient.

Treatment

The administration of CSA and prednisolone (PDN) was initiated simultaneously in all patients a mean of 4.5±5.3 days after admission. PDN and CSA were orally administered at 0.75–1.0 and 3.5–4.5 mg/kg/day, respectively. CSA was administered once a day before breakfast by adjusting the C2, which is correlated with the immunosuppressant effect of CSA, at 1,500 ng/ml or above [20,21]. In this study, the addition of steroid pulse therapy, IVCY, and intravenous immunoglobulin (IVIG) was permitted depending on the disease state.

Clinical findings and laboratory parameters

Patient background, period from the appearance of respiratory symptoms to the beginning of treatment, and contents of treatment were evaluated. The laboratory test items evaluated were creatine kinase, lactic acid dehydrogenase, the creatine kinase/lactic acid dehydrogenase ratio, aldolase, creatinine, C-reactive protein, ferritin, KL-6, antinuclear antibodies, anti-MDA5 and anti-ARS Ab: anti-OJ, anti-EJ, anti-PL-7, anti-PL-12 and anti-Jo-1 Ab. Anti-MDA5 Ab was determined by enzyme-linked immunosorbent assay using recombinant MDA5 antigen (Ori-gene, USA) as described previously [22]. Anti-ARS Ab was determined using a commercially available line blot test kit (Myositis Profile Euroline Blot test kit, Euroimmun, Lübeck, Germany). On blood gas analysis, PaO_2, $PaCO_2$, and the alveolar-arterial oxygen gradient ($P[A-a]O_2$) were evaluated. The $P[A-a]O_2$ was calculated approximately using the following formula:

$$P[A\text{-}a]O_2 = 713 \times FiO_2 - PaCO_2/0.8 - PaO_2$$

Statistical analysis

Statistical analyses were performed with Fisher's exact test or the Mann-Whitney U-test for the comparison of baseline clinical and laboratory findings. Data are presented as the mean ± s.e.m. and were analysed with the statistical program JMP for Windows, version 9.0 (SAS Institute Inc., Cary, NC, USA). To determine the most suitable cut-off level, we used receiver operating characteristic (ROC) curve analysis. To identify prognostic factors, multivariate analyses with a multiple logistic regression model were conducted including the variables with significant differences on univariate analysis. Age- and sex-adjusted Cox regression analysis was performed to establish the prognostic factors. The Kaplan-Meier method was used to assess survival curves and the log-rank test to evaluate the significance of differences between the two groups. A P value of <0.05 was considered to indicate significance.

Results

Outcomes of patients with DM-A/SIP treated by CSA/GC combination therapy

Figure 1 shows the outcomes at 24 weeks after the beginning of early CSA/PDN combination therapy. Of the 32 DM-A/SIP patients, 25 survived and 7 died. Of these 7 patients, 5 died due to exacerbation of DM-A/SIP within 8 weeks after the beginning of treatment. The remaining 2 died due to sepsis and cytomegalovirus infection.

Comparison of clinical and laboratory findings between survivors and dead

Table 1 shows the clinical findings and contents of treatment in the 5 patients who died of DM-A/SIP and the 25 survivors. No significant differences were observed in age, sex, number of C-ADM, or time from the appearance of respiratory symptoms to the beginning of treatment between the surviving patients and those who died. In the 5 patients who died, the mean survival period after the beginning of treatment was 6.2±2.5 weeks, indicating early death ($P<0.001$). Concerning the contents of treatment, there was no difference in the dose of PDN. Although the dose of CSA was significantly higher in those who died compared with the survivors, no difference was observed in the C2 of CSA between

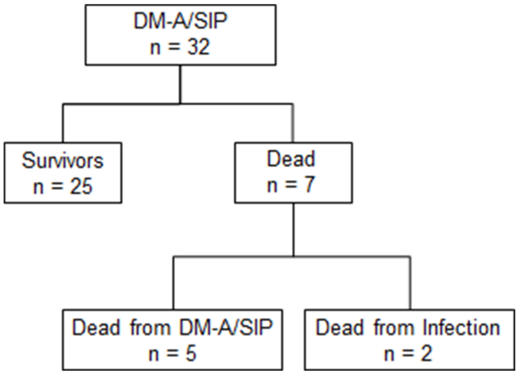

Figure 1. Outcomes at 24 weeks after beginning the cyclosporine A/glucocorticosteroid combination therapy. DM-A/SIP: acute/subacute interstitial pneumonia in dermatomyositis.
doi:10.1371/journal.pone.0089610.g001

the two groups. Also, steroid pulse therapy, IVCY, and IVIG were used significantly more frequently in those who died.

Table 2 shows the laboratory findings from the 2 groups. Among the serum biomarkers, the ferritin level was significantly higher in those who died than in the survivors ($P<0.001$). The KL-6 level was also higher in those who died than in the survivors, but the difference was not significant ($P=0.085$). No difference was observed in the creatine kinase, lactic acid dehydrogenase, the creatine kinase/lactic acid dehydrogenase ratio, aldolase, creatinine, C-reactive protein values between the 2 groups. Although there was no difference in the positive rate of ANA between the 2 groups, all patients positive for anti-ARS Ab survived. No significant differences were noted in PaO_2 or $PaCO_2$, but the P[A-a]O_2 was significantly larger in those who died than in the survivors ($P=0.002$).

Table 1. Clinical characteristics of patients.

	Dead	Survivors	*P* value
No.	5	25	-
Age, yrs	65.6±10.3	57.2±9.5	0.080
Female, n (%)	3 (60.0)	21 (84.0)	*0.254*
C-ADM, n (%)	5 (100)	17 (68.0)	*0.286*
From onset to treatment, months	2.5 (1.0–3.5)	2.5 (1.0–5.5)	*0.832*
PDN, mg/kg/day	1.0 (0–1.0)	1.0 (0.8–1.1)	*0.075*
CSA, mg/kg/day	4.0 (3.8–5.0)	4.0 (3.5–4.5)	*0.046*
CSA trough, ng/ml	191.4 (154.1–456.0)	175.4 (95.0–445.7)	*0.175*
CSA C2, ng/ml	1488 (1262–2003)	1953 (814–2873)	*0.984*
Steroid pulse, n (%)	4 (80.0)	3 (12.0)	*0.006*
IVCY, n (%)	4 (80.0)	10 (40.0)	*0.042*
IVIG, n (%)	3 (60.0)	0 (0.0)	*0.003*
Survival time, weeks	7 (2–8)	24 (24–24)	*<0.001*

Abbreviations: *C-ADM*, clinical amyopathic dermatomyositis; *PDN*, prednisolone; *CSA*, cyclosporine; *C2*, 2-hour postdose blood concentration; *IVCY*, intravenous pulse cyclophosphamide; *IVIG*, intravenous immunoglobulin.
Data are presented as the mean ± standard deviation (SD), median value (range) or number of subjects. P value was estimated by Fisher's exact test or Mann-Whitney U-test.
doi:10.1371/journal.pone.0089610.t001

Table 2. Pre-treatment laboratory findings of patients.

	Dead	Survivors	*P* value
No.	5	25	-
CK, U/l	139 (68–434)	232 (41–13574)	*0.327*
LD, U/l	448 (272–485)	381 (181–1463)	*0.632*
CK/LD	0.5 (0.2–0.9)	0.7 (0.1–9.5)	*0.221*
ALD, U/l	10.3 (5.2–12.9)	9.3 (3.5–205.6)	*0.405*
Cr, mg/dl	0.46 (0.34–1.00)	0.53 (0.41–0.98)	*0.753*
CRP, mg/dl	1.64 (0.21–6.49)	0.66 (0.04–9.72)	*0.692*
Ferritin, ng/ml	1611 (1013–2376)	133 (28–1102)[a]	*<0.001*
KL-6, U/ml	1329 (512–3789)	1064 (206–2620)	*0.085*
ANA, n (%)	3 (60.0)	16 (69.6)	*1.000*
Cytoplasmic staining, n(%)	0 (0.0)[b]	8 (50.0)[a]	-*
Anti-ARS antibodies, n (%)	0 (0.0)[c]	11 (68.8)[a]	-*
Anti-MDA5 antibodies, n (%)	2 (100)[c]	2 (18.1)[d]	-*
PaO_2, Torr	65.2 (51.8–101.7)	70.2 (50.5–97.6)	*0.985*
$PaCO_2$, Torr	37.5 (29.0–40.0)	38.1 (32.1–67.6)	*0.285*
PaO_2/FiO_2 ratio	281 (245–348)	325 (186–464)	*0.062*
P[A-a]O_2, Torr	83.5 (46.0–432.5)	28.5 (4.8–61.3)	*0.001*

Abbreviations: *CK*, creatine kinase; *LD*, lactic acid dehydrogenase; *ALD*, aldolase; *Cr*, creatinine; *CRP*, C-reactive protein; *ANA*, antinuclear antibodies; ARS, aminoacyl tRNA synthetase; MDA5, melanoma differentiation-associated gene 5; *P[A-a]O_2*, alveolar-arterial oxygen gradient.
Data are presented as the median values (range) or number of subjects. P value was estimated by Fisher's exact test or Mann-Whitney U-test. [a]Number of subjects = 16. [b]Number of subjects = 3. [c]Number of subjects = 2. [d]Number of subjects = 11.
*Statistical analysis was not performed because of small samples.
doi:10.1371/journal.pone.0089610.t002

Extraction of prognostic factors

By univariate analysis of the 25 survivors and the 5 who died, ferritin and the P[A-a]O_2 were suggested as prognostic factors. As a result of multivariate analyses with a multiple logistic regression model, ferritin and P[A-a]O_2 were found to be independent prognostic factors of poor outcome in DM-A/SIP patients treated with early CSA/PSL combination therapy (table 3).

Table 3. Hazard ratios (per unit) of prognostic factors in acute/subacute interstitial pneumonia in dermatomyositis.

Prognostic factor	Hazard ratio	95% CI	P-value
Ferritin	1.003	1.001–1.005	0.002
P[A-a]O_2	1.045	1.017–1.085	0.017

Age- and sex-adjusted Cox regression analysis was performed to establish the prognostic factors.
doi:10.1371/journal.pone.0089610.t003

Cut-off values of ferritin and P[A-a]O_2 and survival rate

To determine cut-off points effective for the prognosis of DM-A/SIP, ROC curve analysis was carried out on ferritin and the P[A-a]O_2. The values that maximised the area under the ROC curve were 595.7 ng/ml for ferritin (sensitivity: 87.5%, specificity: 100%) and 41.9 Torr for P[A-a]O_2 (sensitivity: 91.7%, specificity: 100%). From these results, a ferritin level of ≥600 ng/ml and a P[A-a]O_2 of ≥45 Torr were determined as cut-off values for a poor prognosis. The patients were then divided into 2 groups on the basis of these cut-off values, and Kaplan-Meier survival curves were plotted (Figures 2 and 3). The survival rate after 24 weeks was significantly lower in patients with a level of ferritin of ≥ 600 ng/ml (survival rate: 28.5%) than in those with <600 ng/ml (100%) ($P<0.001$). The survival rate was also significantly lower in patients with a P[A-a]O_2 of ≥45 Torr (survival rate: 28.5%) than in those with a P[A-a]O_2 of <45 Torr (100%) ($P<0.001$).

Survival rate by number of poor prognostic factors

Table 4 shows the number of these prognostic factors observed in each of the survivors and those who died. The number was significantly higher in those who died than in the survivors ($P<0.001$). Figure 4 shows Kaplan-Meier survival curves by the number of prognostic factors. Whereas all patients who had both factors died within 8 weeks, no patient with neither or only 1 of the factors died, with a clear difference in the survival rate ($P<0.001$).

Discussion

We evaluated prognostic factors in DM-A/SIP patients treated using early CSA/PDN combination therapy. Similar to the findings of previous reports, the ferritin level at the beginning of therapy was higher in those who died of DM-A/SIP than in those who survived, and the outcome was poor at ferritin levels of ≥ 600 ng/ml [9,13]. Also, the P[A-a]O_2 was increased more in those who died than in the survivors, and the prognosis was poor at a P[A-a]O_2 of ≥45 Torr. A ferritin level of ≥600 ng/ml and P[A-a]O_2 of ≥45 at the beginning of treatment were independent prognostic factors in DM-A/SIP patients, and the outcome was significantly poorer in patients with both rather than 1 or none of these factors.

The production of ferritin is increased by the activation of reticuloendothelial cells, particularly macrophages. An elevation of the ferritin level in DM-A/SIP patients is considered to indicate the activation of alveolar macrophages and reflects inflammation of and damage to the lungs [9,13]. Ferritin has been reported as a prognostic factor in DM-A/SIP patients. Gono et al. reported that the serum ferritin level was markedly increased in DM-A/SIP patients and that the survival rate was significantly lower in patients with a ferritin level of ≥1,500 ng/ml [9]. Moreover, they reported that the serum ferritin level was increased in DM-A/SIP patients positive for anti-MDA5 Ab and was related to the prognosis and disease activity [8]. In our study also, the outcome was poor when the ferritin level at the beginning of treatment was elevated to ≥600 ng/ml. The ferritin level that exacerbated the prognosis differed compared with that in previous reports, but this discrepancy is considered to have been due to the limited number of subjects, time of ferritin measurement, patients' background, and treatment contents.

The P[A-a]O_2 is a simple index of the pulmonary diffusion capacity and is known to increase in IP patients due to interstitial proliferation [23]. In this study, the P[A-a]O_2 as well as ferritin level at the beginning of treatment were found to be prognostic factors of DM-A/SIP with early CSA/PDN combination therapy. The P[A-a]O_2 was reportedly increased in anti-MDA5 antibody-positive patients who died due to rapidly progressing DM-IP compared with survivors [9]. In the acute exacerbation of DAD and interstitial pneumonia, pulmonary diffusion is severely impaired by fibrosis of the alveolar matrix or by inflammatory cell infiltration. On respiratory function testing, the degree of gas transfer reduction is reflected by the decrease in the diffusing

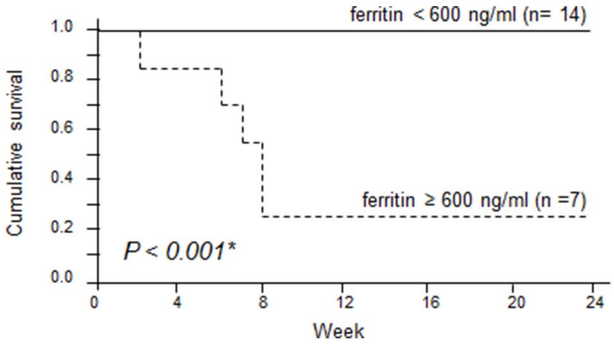

Figure 2. Survival curves of patients based on their pre-treatment serum ferritin levels. Survival rates were calculated by the Kaplan-Meier test and compared by log-rank test. (solid line: <600 ng/ml; dashed line: ≥600 ng/ml).
doi:10.1371/journal.pone.0089610.g002

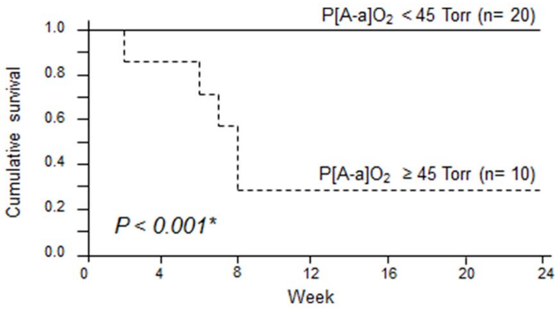

Figure 3. Survival curves of patients based on their pre-treatment P[A-a]O_2 value. Survival rates were calculated by the Kaplan-Meier test and compared by log-rank test. (solid line: <45 Torr; dashed line: ≥45 Torr).
doi:10.1371/journal.pone.0089610.g003

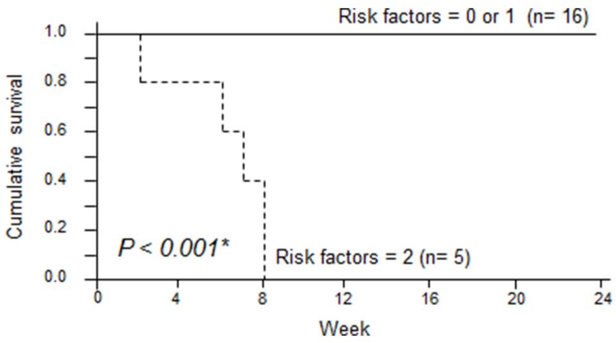

Figure 4. Survival curves of patients based on their pre-treatment number of poor prognostic factors. Survival rates were calculated by the Kaplan-Meier test and compared by log-rank test. (solid line: 0 or 1 factor; dashed line: 2 factors).
doi:10.1371/journal.pone.0089610.g004

Table 4. Number of prognostic factors and survival rate in the study patients.

No. of prognostic factors	Dead (n = 5)	Survivors (n = 16)	Survival rate (%)
0	0	13	100
1	0	3	100
2	5	0	0

The number of subjects $P<0.001$.
doi:10.1371/journal.pone.0089610.t004

capacity of the lungs for carbon monoxide, which is also reduced in DM-IP [11,12]. However, in patients with severe DM-A/SIP, accurate pulmonary function testing is impossible due to complications such as dyspnea and pneumomediastinum. Therefore, the P[A-a]O_2 is simple and prognostically useful.

In the DM-A/SIP patients treated by early CSA/PDN combination therapy, ferritin ≥600 ng/ml and P[A-a]O_2 ≥45 Torr were independent prognostic factors, and all patients with both of these factors died. In these patients, the outcome could not be improved even by additional treatments such as IVCY and IVIG. Recently, mycophenolate mofetil and blood purification therapy have been reported to be effective in patients with refractory DM-A/SIP [24–26]. In patients with a poor prognosis, immunosuppressant therapy such as IVCY and mycophenolate mofetil and blood purification therapy should be considered from the beginning of treatment in addition to early CSA/PDN combination therapy.

The blood KL-6 level, which increases due to the excessive expression of pulmonary alveolar type II epithelial cells and an increase in alveolar vascular permeability, is a biomarker used for the diagnosis and evaluation of IP. In patients with IP including DM, the prognosis was reported to be exacerbated when the KL-6 level was ≥1,000 U/l [14]. However, regarding DM-IP in particular, there have been no reports that the pre-treatment KL-6 level is related to disease severity or outcome. There was no difference in KL-6 level between those who died due to DM-A/SIP and the survivors in this study. Arai et al. reported that the KL-6 level 2–4 weeks after the beginning of treatment was markedly elevated in those who died due to DM-IP compared with those who survived [27]. The clinical course of DM-A/SIP is rapidly progressive and this biomarker may not reflect the disease activity in rapid course of interstitial pneumonia.

This study was carried out retrospectively in a small number of patients. In addition, autoantibodies and lung high-resolution computed tomography findings were not evaluated in all enrolled patients or in a large number of patients. Prognostic factors associated with the disease must still be extracted by evaluating a larger number of patients.

Conclusion

We showed that increase of the serum ferritin level and P[A-a]O_2 are prognostic factors in DM-A/SIP patients and that the outcome is exacerbated when these factors are present. Serum ferritin and P[A-a]O_2 are easy and readily-available screening tools. This study provides a simple and low-cost approach to risk-stratify DM-A/SIP patients upon admission thereby assisting clinicians in evaluating which patients may benefit from more aggressive initial immunosuppressive therapy.

Author Contributions

Conceived and designed the experiments: KI TK TT. Performed the experiments: KI. Analyzed the data: KI KH TI. Contributed reagents/materials/analysis tools: KI TS SY YK SM TH. Wrote the paper: KI.

References

1. Tazelaar HD, Viggiano RW, Pickersgill J, Colby TV (1990) Interstitial lung disease in polymyositis and dermatomyositis. Clinical features and prognosis as correlated with histologic findings. Am Rev Respir Dis 141: 727–733.
2. Miyazaki E, Ando M, Muramatsu T, Fukami T, Matsuno O, et al. (2007) Early assessment of rapidly progressive interstitial pneumonia associated with amyopathic dermatomyositis. Clin Rheumatol 26: 436–439.
3. Kotani T, Makino S, Takeuchi T, Kagitani M, Shoda T (2008) Early intervention with corticosteroids and cyclosporin A and 2-hour postdose blood concentration monitoring improves the prognosis of acute/subacute interstitial pneumonia in dermatomyositis. J Rheumatol 35: 254–259.
4. Kameda H, Nagasawa H, Ogawa H, Sekiguchi N, Takei H, et al. (2005) Combination therapy with corticosteroids, cyclosporin A, and intravenous pulse cyclophosphamide for acute/subacute interstitial pneumonia in patients with dermatomyositis. J Rheumatol 32: 1719–1726.
5. Ando M, Miyazaki E, Yamasue M, Sadamura Y, Ishii T, et al. (2010) Successful treatment with tacrolimus of progressive interstitial pneumonia associated with amyopathic dermatomyositis refractory to cyclosporine. Clin Rheumatol 29: 443–445.
6. Mimori T, Nakashima R, Hosono Y (2012) Interstitial lung disease in myositis: clinical subsets, biomarkers, and treatment. Curr Rheumatol Rep 14: 264–274.
7. Koreeda Y, Higashimoto I, Yamamoto M, Takahashi M, Kaji K, et al. (2010) Clinical and pathological findings of interstitial lung disease patients with anti-aminoacyl-tRNA synthetase autoantibodies. Intern Med 49: 361–369.
8. Gono T, Kawaguchi Y, Satoh T, Kuwana M, Katsumata Y, et al. (2010) Clinical manifestation and prognostic factor in anti-melanoma differentiation-associated gene 5 antibody-associated interstitial lung disease as a complication of dermatomyositis. Rheumatology (Oxford) 49: 1713–1719.
9. Gono T, Sato S, Kawaguchi Y, Kuwana M, Hanaoka M, et al. (2012) Anti-MDA5 antibody, ferritin and IL-18 are useful for the evaluation of response to treatment in interstitial lung disease with anti-MDA5 antibody-positive dermatomyositis. Rheumatology (Oxford) 51: 1563–1570.
10. Hayashi S, Tanaka M, Kobayashi H, Nakazono T, Satoh T, et al. (2008) High-resolution computed tomography characterization of interstitial lung diseases in polymyositis/dermatomyositis. J Rheumatol 35: 260–269.
11. White B, Moore WC, Wigley FM, Xiao HQ, Wise RA (2000) Cyclophosphamide is associated with pulmonary function and survival benefit in patients with scleroderma and alveolitis. Ann Intern Med 132: 947–954.
12. Yamasaki Y, Yamada H, Yamasaki M, Ohkubo M, Azuma K, et al. (2007) Intravenous cyclophosphamide therapy for progressive interstitial pneumonia in patients with polymyositis/dermatomyositis. Rheumatology (Oxford) 46: 124–130.
13. Gono T, Kawaguchi Y, Hara M, Masuda I, Katsumata Y, et al. (2010) Increased ferritin predicts development and severity of acute interstitial lung disease as a complication of dermatomyositis. Rheumatology (Oxford) 49: 1354–1360.
14. Satoh H, Kurishima K, Ishikawa H, Ohtsuka M (2006) Increased levels of KL-6 and subsequent mortality in patients with interstitial lung diseases. J Intern Med 260: 429–434.
15. Bohan A, Peter JB (1975) Polymyositis and dermatomyositis (first of two parts). N Engl J Med 292: 344–347.
16. Bohan A, Peter JB (1975) Polymyositis and dermatomyositis (second of two parts). N Engl J Med 292: 403–407.
17. Sontheimer RD (2002) Dermatomyositis: an overview of recent progress with emphasis on dermatologic aspects. Dermatol Clin 20: 387–408.
18. Gerami P, Schope JM, McDonald L, Walling HW, Sontheimer RD (2006) A systematic review of adult-onset clinically amyopathic dermatomyositis (dermatomyositis siné myositis): a missing link within the spectrum of the idiopathic inflammatory myopathies. J Am Acad Dermatol 54: 597–613.

19. American Thoracic Society (2000) Idiopathic pulmonary fibrosis: diagnosis and treatment. International consensus statement. American Thoracic Society (ATS), and the European Respiratory Society (ERS). Am J Respir Crit Care Med 161: 646–664.
20. Nagai K, Takeuchi T, Kotani T, Hata K, Yoshida S, et al. (2011) Therapeutic drug monitoring of cyclosporine microemulsion in interstitial pneumonia with dermatomyositis. Mod Rheumatol 21: 32–36.
21. Levy G, Thervet E, Lake J, Uchida K, Consensus on Neoral C(2): Expert Review in Transplantation (CONCERT) Group (2002) Patient management by Neoral C(2) monitoring: an international consensus statement. Transplantation 73: S12–18.
22. Chen F, Wang D, Shu X, Nakashima R, Wang G (2012) Anti-MDA5 antibody is associated with A/SIP and decreased T cells in peripheral blood and predicts poor prognosis of ILD in Chinese patients with dermatomyositis. Rheumatol Int 32: 3909–3915.
23. Heikkilä HP, Lappalainen AK, Day MJ, Clercx C, Rajamäki MM (2011) Clinical, bronchoscopic, histopathologic, diagnostic imaging, and arterial oxygenation findings in West Highland White Terriers with idiopathic pulmonary fibrosis. J Vet Intern Med 25: 433–439.
24. Morganroth PA, Kreider ME, Werth VP (2010) Mycophenolate mofetil for interstitial lung disease in dermatomyositis. Arthritis Care Res (Hoboken) 62: 1496–1501.
25. Kakugawa T, Mukae H, Saito M, Ishii K, Ishimoto H, et al. (2008) Rapidly progressive interstitial pneumonia associated with clinically amyopathic dermatomyositis successfully treated with polymyxin B-immobilized fiber column hemoperfusion. Intern Med 47: 785–790.
26. Ichiyasu H, Horio Y, Tsumura S, Hirosako S, Sakamoto Y, et al. (2012) Favorable outcome with hemoperfusion of polymyxin B-immobilized fiber column for rapidly progressive interstitial pneumonia associated with clinically amyopathic dermatomyositis: report of three cases. Mod Rheumatol, in press.
27. Arai S, Kurasawa K, Maezawa R, Owada T, Okada H, et al. (2013) Marked increase in serum KL-6 and surfactant protein D levels during the first 4 weeks after treatment predicts poor prognosis in patients with active interstitial pneumonia associated with polymyositis/dermatomyositis. Mod Rheumatol 23: 872–883.
